# Empiric Usage of “Anti-Pseudomonal” Agents for Hospital-Acquired Urinary Tract Infections

**DOI:** 10.3390/antibiotics11070890

**Published:** 2022-07-04

**Authors:** Ori Rahat, Murad Shihab, Elhai Etedgi, Debby Ben-David, Inna Estrin, Lili Goldshtein, Shani Zilberman-Itskovich, Dror Marchaim

**Affiliations:** 1 Unit of Infection Control, Shamir (Assaf Harofeh) Medical Center, Zerifin 7030000, Israel; shihab-murad@hotmail.com (M.S.); elhaietedgi@gmail.com (E.E.); innae@shamir.gov.il (I.E.); lilig@shamir.gov.il (L.G.); shani.zilberman@mail.huji.ac.il (S.Z.-I.); drormarchaim@shamir.gov.il (D.M.); 2 Sackler School of Medicine, Tel-Aviv University, Tel-Aviv 6997801, Israel; debbyb@wmc.gov.il; 3 Unit of Infection Control, Wolfson Medical Center, Holon 5822012, Israel

**Keywords:** UTI, healthcare-associated infections, stewardship, HAUTI, CAUTI, anti-Pseudomonal agents, *Pseudomonas aeruginosa*

## Abstract

Hospital-acquired urinary tract infection (HAUTI) is one of the most common hospital-acquired infections, and over 80% of HAUTI are catheter-associated (CAUTI). *Pseudomonas aeruginosa*, as well as other non-glucose fermenting Gram negative organisms (NGFGN, e.g., *Acinetobacter baumannii*), are frequently covered empirically with “anti-Pseudomonals” being administered for every HAUTI (and CAUTI). However, this common practice was never trialed in controlled settings in order to quantify its efficacy and its potential impacts on hospitalization outcomes. There were 413 patients with HAUTI that were included in this retrospective cohort study (2017–2018), 239 (57.9%) had CAUTI. There were 75 NGFGN infections (18.2% of HAUTI, 22.3% of CAUTI). *P. aeruginosa* was the most common NGFGN (82%). Despite multiple associations per univariable analysis, recent (3 months) exposure to antibiotics was the only independent predictor for NGFGN HAUTI (OR = 2.4, CI-95% = 1.2–4.8). Patients who received empiric anti-Pseudomonals suffered from worse outcomes, but in multivariable models (one for each outcome), none were independently associated with the empiric administration of anti-Pseudomonals. To conclude, approximately one of every five HAUTI (and CAUTI) are due to NGFGN, which justifies the practice of empiric anti-Pseudomonals for patients with HAUTI (and CAUTI), particularly patients who recently received antibiotics. The practice is not associated with independent deleterious impacts on outcomes.

## 1. Introduction

Hospital-acquired infections (HAI) constitute a significant and common nosocomial complication [1,2]. Hospital-acquired urinary tract infections (HAUTI) account for 20% of HAI in the U.S. and 24% in Europe [1]. Approximately 80–97% of HAUTI are classified as catheter-associated UTI (CAUTI) [3]. HAUTI (both CAUTI and non-CAUTI HAUTI) have numerous predictors, among them are age over 50 years, diabetes, chronic renal failure, female gender, institutionalization, the duration in which the catheter is in place, non-aseptic technique that is used for catheter insertion, and failure to conduct daily rounds in the unit pertaining to the appropriateness of catheter removal [4,5].

Most HAUTI (including CAUTI) are caused by enteric pathogens, i.e., Enterobacterales (e.g., *Escherichia coli*, *Klebsiella pneumoniae*) and enterococci species [6,7]. Additional groups of nosocomial pathogens that can cause HAUTI (and specifically CAUTI [8]), are the non-glucose fermenting Gram negatives (NGFGN): e.g., *Pseudomonas aeruginosa*, *Acinetobacter baumannii* [8,9]. These opportunistic nosocomial pathogens account for 15% of HAI according to some series [8], and their epidemiological significance is derived primarily from their inherent resistance to many antimicrobial agents [10], and their potential to create biofilms and difficult-to-treat infections, particularly in the presence of foreign devices (e.g., catheter, nephrostomy, stent) [10,11]. These NGFGN pathogens necessitate the administration of broad-spectrum agents, which are commonly referred to as “anti-Pseudomonals” (e.g., piperacillin, ceftazidime, cefepime, meropenem, aminoglycosides, fluoroquinolones), since *P. aeruginosa* is the commonest NGFGN in many HAUTI series [12,13].

Some prescribers administer empirically anti-Pseudomonals for every HAUTI (including every CAUTI) [14], since delaying administration of appropriate antimicrobial therapy (DAAT) is the strongest modifiable predictor for mortality in severe sepsis [15]. However, this practice is not scientifically supported. Wide empiric usage of broad-spectrum anti-Pseudomonals might be associated with fiscal and ecological deleterious impacts, both on individual patients and on health institutions in general. Possible deleterious outcomes might be superfluous side effects, acquisitions of multi-drug-resistant organisms (MDRO) carriage, or acute *Clostridioides difficile* infections (CDI) [16,17]. Moreover, for certain severe infectious syndromes that are caused by non-NGFGN-susceptible organism, administering a narrowed-spectrum agent is frequently more effective, bactericidal, and safer, in comparison to broader-spectrum anti-Pseudomonals [18]. Therefore, due to these complexities, the majority of professional societies, including the Infectious Disease Society of America [12], and the European Society for Clinical Microbiology and Infectious Diseases [19], currently avoid direct recommendations pertaining to uniform empiric administration of anti-Pseudomonals for every HAUTI (or for every CAUTI), and suggest to base this practice on the local epidemiology [20,21].

Some of the anti-Pseudomonals that are administered empirically for HAUTI, are beta-lactams (e.g., piperacillin/tazobactam, ceftazidime, ceftazidime-avibactam, ceftolozane-tazobactam, meropenem, imipenem) and some are non-beta-lactam classes (e.g., aminoglycosides, fluoroquinolones, polymyxins). These non-beta-lactam anti-Pseudomonals, frequently cover Enterobacterales as well and, therefore, could be administered empirically in HAUTI to cover both NGFGN and Enterobacterales (some cover also enterococci to a certain degree). Therefore, it is frequently complicated, while reviewing a case retrospectively, to determine whether the prescriber intended to administer the non-beta-lactam anti-Pseudomonal agent, in order to cover specifically NGFGN, or to cover both NGFGN and Enterobacterales. Among a cohort of patients who received solely beta-lactam agents, it is easier to differentiate patients for which the prescriber deliberately wanted to cover NGFGN, as he can prescribe a beta-lactam with/out anti-Pseudomonal coverage. Our study aim was, therefore, to conduct a comprehensive epidemiological investigation, analyzing this practice of prescribing empiric anti-Pseudomonals for every HAUTI (and every CAUTI).

## 2. Results

The study included 413 patients with HAUTI, of which 239 (58%) were CAUTI. A total of 75 patients (18.2%) had NGFGN HAUTI. A total of 41 patients (10%) had a chronic urinary catheter. The study population was primarily composed of elderly (*n* = 340, 82.3%) women (*n* = 233, 56.4%), with multiple underlying co-morbidities and background conditions (i.e., median Charlson’s combined condition score [22] of 6, IQR = 5–8). The burden of recent healthcare exposures were substantial among the affected population, i.e., 76 (18.5%) patients were chronic long-term care facility (LTCF) residents, 144 (34.9%) had previous recent (3 months) hospitalization at acute-care facility, and nearly half (*n* = 203, 49%) were recently (3 months) exposed to antimicrobial regimens. The median time from admission to HAUTI diagnosis was five days. Of all HAUTI, 42 (10.2%) had concurrent bloodstream infection and 37 (9%) developed septic shock during the index event. With regards to HAUTI outcomes, 110 (26.6%) patients died during the index hospitalization and 159 (38.5%) died within 90 days (i.e., all-cause mortality). Among the survivors of the index hospitalization (*n* = 303), the median stay at the hospital from the day of HAUTI diagnosis to discharge was 9 days, 145 (48%) experienced functional status [23] deterioration, 109 (40%) patients who were admitted from home to the index hospitalization where eventually discharged to LTCFs, 15 (4.3%) developed acute CDI, and 109 (36%) had additional hospitalization/s in the following three months.

In Table 1, the list of pathogens causing HAUTI is depicted.

One of every four patients (i.e., 107 patients, 26%) had a polymicrobial HAUTI. *E. coli* was the commonest pathogen, followed by *Enterococcus* species and *K. pneumoniae*. *Pseudomonas aeruginosa* was the most common NGFGN (i.e., 82%), followed by *A. baumannii* (16%), which was the most common NGFGN causing extensively drug-resistant organism (XDRO) HAUTI (48%). There were 126 Enterobacterales isolates (30%), displaying phenotypically an extended-spectrum beta-lactamase (ESBL) and/or hyper AmpC production.

### 2.1. NGFGN HAUTI-Risk Factors and Outcomes

Table 2 depicts the univariable analyses of NGFGN HAUTI predictors and outcomes.

There were no significant age differences in comparison to patients with HAUTI resulting from non-NGFGN pathogens. However, NGFGN HAUTI, in reverse to the gender composition of the entire cohort, were predominantly diagnosed among men, and it was diagnosed considerably later into the hospitalization (i.e., additional five days in comparison to non-NGFGN HAUTI). There were no significant differences in the pre-HAUTI functional or cognitive statuses, and the severity of background co-morbidities indexes were similar between the groups (e.g., Charlson’s scores [22]). However, patients with NGFGN HAUTI had significant additional recent exposures to healthcare. Patients were significantly more often flagged as known recent MDRO carriers (from the past two years), had recent (i.e., previous three months) documentation of (1) hospitalizations, (2) invasive procedures, (3) presence of chronic invasive devices (mainly urinary catheters), and (4) exposures to antimicrobials.

The presence of a catheter at HAUTI diagnosis, and the duration in which the catheter was in place both impacted significantly the probability for NGFGN HAUTI, translating to significantly higher proportions of CAUTIs (71% of NGFGN HAUTI vs. 55% of non-NGFGN HAUTI, *p* = 0.01). The severity of acute illness indices were similar between patients with NGFGN vs. patients with other pathogens. Patients with NGFGN suffered from additional stay (in days) at the acute-care facility following their HAUTI, but the other clinical outcomes were not significantly worse, with no enhanced mortality rates, or disability and morbidity sequels that were enhanced among survivors of the index hospitalization. Next, we constructed a multivariable model of predictors for NGFGN HAUTI. Despite multiple possible predictors per univariable analyses (as depicted in bold in Table 2), recent (past 3 months) exposure to antibiotics remained the only independent predictor for NGFGN HAUTI (aOR = 2.4 [CI-95% 1.2–4.8], *p* = 0.01).

### 2.2. The Empiric Usage of Anti-Pseudomonal Agents

There were only 204 (51.1%) patients who received an “appropriate” antimicrobial agent within 48 h (per in vitro susceptibility results). Of the total population (*n* = 413), there were 181 (44.5%) patients who received an empirical regimen that contained anti-Pseudomonals: i.e., 110 (61%) received anti-Pseudomonal beta-lactam, 62 (34%) received fluoroquinolone, 16 (9%) received aminoglycoside, and two (1.1%) received colistin. Among the population who received anti-Pseudomonals empirically (*n* = 181), the time for initiation of appropriate therapy was significantly shorter in comparison to patients who did not receive empirically a regimen that contained anti-Pseudomonals (*n* = 232, *p* < 0.001). In addition, eventual NGFGN HAUTI was significantly more common among patients who received empiric anti-Pseudomonals (OR = 1.7, CI-95% = 1.1–2.8). With regards to the catheter indication, which was documented among 345 patients, post-surgery was a “risk factor” for receiving empiric anti-Pseudomonals (OR = 2.1, CI-95% = 1.01–4), while acute urinary retention was a “protecting factor”, i.e., implying the majority of patients with this indication for acute catheterization did not receive empiric anti-Psudomonals, but narrower-spectrum agents (OR = 0.4, CI-95% = 0.2–0.9). Other baseline characteristics, i.e., demographics, background co-morbidities, Charlson’s indexes [22], baseline functional and cognitive statuses, recent healthcare exposures, and acute illness indices, did not differ between the groups. The hospitalization’s outcomes did not differ between the groups as well (data not shown).

Next, with the rationale that is depicted in methods, we analyzed the epidemiology and impacts of empiric anti-Pseudomonal administration only among the patients who received a beta-lactam-only regimen (*n* = 199). Among this group (Table 3), it is easier to analyze the predictors and outcomes that are associated more directly with the practice of empiric anti-Pseudomonal prescription.

As depicted in Table 3, in contrast to the same analysis that was executed among the entire cohort, there were multiple risk factors and devastating outcomes that were associated with empiric anti-Pseudomonals administration, while analyzed only among the cohort of patients that received a beta-lactam-only regimen. The patients who received anti-Pseudomonal beta-lactams were those who were diagnosed with HAUTI later into the hospitalization, and NGFGN HAUTI was eventually diagnosed significantly more often (analyzed among the microbiologically-confirmed cases only). Patients with empiric anti-Pseudomonal beta-lactam coverage were also more dependent in terms of their background functional status [23], but the Charlson’s indexes [22] were similar between the groups. The frequency of recent hospitalization/s and of recent exposures to antimicrobials, was significantly elevated among these patients. Despite the fact that the portion of patients with a urinary catheter at HAUTI diagnosis was similar, the number of catheter days prior to HAUTI and the portion of CAUTI cases was significantly elevated among HAUTI patients who received empirically anti-Pseudomonal beta-lactams. The severity of acute illness indices were similar with similar rates of severe sepsis or septic shock [25], but anti-Pseudomonals were more often prescribed to patients with HAUTI that was diagnosed at ICUs and among patients with documented fever. In multivariable model, the only independent predictors to receive anti-Pseudomonal coverage, among patients who were manage with beta-lactam agents only, were fever (aOR = 4.0, *p* < 0.001) and rapidly fatal condition per McCabe score (aOR = 2.1, *p* = 0.04) [26] at the day of culture.

With regards to HAUTI outcomes (bottom of Table 3), patients with empiric anti-Pseudomonal beta-lactam coverage died more often during the index hospitalization, but the overall 90-day survival rates were similar between groups. The total length of stay was also longer among this group of patients, but when the length of stay was analyzed only from the HAUTI diagnosis to discharge and only among survivors of the index hospitalization, it was not significantly elongated. Patients who received anti-Pseudomonals empirically at HAUTI diagnosis and survived the index hospitalization, experienced more often functional status deterioration [23], additional hospitalization/s in the following 3 months, and among those who were admitted from home, more patients were eventually discharged to LTCF following deconditioning during their stay. In separate multivariable models, however, one for each of these aforementioned outcome, anti-Pseudomonal coverage did not remain independently associated with any worse outcomes (data not shown).

## 3. Discussion

HAUTI is a serious infection that is associated with detrimental outcomes to patients [1,2]. In some countries, including in Israel, HAUTI rates are mandatorily reported to health authorities, to the general public, and are used to prioritize fiscal support to health institutions (i.e., as ‘pay-per-performance’ measures) [28]. HAUTI and CAUTI are often caused by MDRO, primarily Gram negatives, which impose an additional burden and threat to patients and health facilities [8]. NGFGN are common MDRO in some regions, and they necessitate the administration of specified agents, i.e., “anti-Pseudomonal” agents, which are frequently given empirically to every patient with HAUTI and to every patient with CAUTI [12]. This practice of broad empiric usage for every HAUTI patient is widely accepted [14], although it is not directly recommended by professional societies and it was not yet studied in a scientifically controlled trial, which captures the short-term and the longer-term consequences of this practice.

In this study we queried and analyzed the epidemiology of 413 patients with HAUTI, of which 239 (58%) had CAUTI. NGFGN were common among this cohort of HAUTI cases, i.e., 18.2% of HAUTI and 22.3% of CAUTI (Table 1), which might justify the ‘non-formal’ recommendation to treat empirically with anti-Pseudomonals every HAUTI and particularly every CAUTI. Despite multiple possible predictors per univariable analysis (Table 2), NGFGN HAUTI were eventually independently associated only with recent exposure to (any) antibiotics (aOR = 2.4, CI-95% 1.2–4.8). Therefore, based on this study and others [20], we recommend that among patients with recent exposure to antibiotics, HAUTI should be managed empirically with anti-Pseudomonals. However, anti-Pseudomonals were empirically administered in this study to less than half of the patients (i.e., 45%), and an appropriate (per in vitro susceptibility) antimicrobial agent in general was administered in less than 48 h (by which time the urine culture results are usually available), only to 51% of patients. This reflects the commonality of inappropriate antimicrobial management of HAUTI in hospitals. This has been reported in additional studies [29]. Moreover, HAUTI is sometimes perceived as a ‘milder’ infectious syndrome [30], but 27% of the patients that were included in this cohort of patients with HAUTI had died during the index hospitalization, and 39% had died within three months. This further highlights the epidemiological significance of this clinical entity, and the importance of effective therapeutic management in accordance to controlled scientific data and coupled with following appropriate antimicrobial stewardship practices.

In order to explore the empiric practices of prescribers for HAUTI, we focused specifically on patients who were managed with beta-lactam-only agents (Table 3), since among this cohort, it is easier to explore the features that are associated specifically with the empirical administration of anti-Pseudomonals. Despite multiple significant associations per univariable analysis, in multivariable model, the eventual independent predictors for empiric anti-Pseudomonal administration were high fever (aOR = 4.0, *p* < 0.001) and rapidly fatal condition per McCabe score (aOR = 2.1, *p* = 0.04) [26] at the day of culture, i.e., implying that the only independent predictors for anti-Pseudomonal empiric administration were severer indices of acute illness, while the other potential risk factors per univariable analysis (Table 3), proved all to be confounders, not true predictors for anti-Pseudomonal administration. With regards to HAUTI outcomes, anti-Pseudomonal coverage was associated with several worse outcomes, but in separate multivariable models, empiric anti-Pseudomonals administration was not independently associated with any favorable nor worse outcome. Therefore, since NGFGN HAUTI is relatively common (~20%), and empiric administration of anti-Pseudomonals was not independently associated with worse outcome in sub-group analysis of patients that were managed with beta-lactam only agents, we support the current practices of empiric anti-Pseudomonal administration that are executed in many centers, specifically among patients who recently received antimicrobials, and specifically among patients with severer indices of acute illness. For patients with milder disease, managing empirically the infection (for the first two days until microbiological diagnosis) without anti-Pseudomonals was not associated with any worse outcomes as well. Non-beta-lactam anti-Pseudomonals, which covers Enterobacterales as well (e.g., aminoglycosides, fluoroquinolones), could be an alternative management option, depending on the local epidemiology of circulating NGFGN strains causing HAUTI.

Our study has several limitations and inherent biases that are associated with its retrospective chart-review-based design that was executed at a single center. However, conducting prospective multicenter comparative trial in this research field seems ethically implausible in light of the results that are presented herein and elsewhere [12]. Therefore, this relatively big retrospective study (413 HAUTI patients), with analyses pertaining specifically to prescription practices, could provide ‘real-world’ controlled data pertaining to empiric administration of anti-Pseudomonals for every HAUTI and CAUTI.

## 4. Conclusions

Approximately one of every five HAUTI (and CAUTI) are due to NGFGN, which justifies the practice of empiric anti-Pseudomonals administration for patients with HAUTI (and CAUTI), particularly for patients who recently received antibiotics. The practice is not associated with independent deleterious impacts on outcomes. It is necessary to conduct future prospective trials to quantify the impact of this practice on various clinical and fiscal outcomes.

## 5. Materials and Methods

A retrospective cohort study was conducted at Shamir (Assaf Harofeh) Medical Center (SMC), central Israel, for calendar years 2017–2018. HAUTI and CAUTI were determined in accordance to the surveillance definitions of the Centers for Disease Control and Prevention (CDC) [27]. The local ethics (“Helsinki”) committee at SMC had approved the study prior its initiation.

The study included adult patients (>18 years) with HAUTI, both CAUTI and non-CAUTI HAUTI patients [27]. Patients with asymptomatic bacteriuria per established definition [27] were excluded. Every patient was included in the analysis only once (i.e., “patient-unique” episodes). The data were extracted from all available records, including demographic parameters, background illnesses and conditions, recent exposures to healthcare (i.e., to settings, environments, procedures), parameters that are associated with the presence of a urine catheter, acute illness indices, and various clinical outcomes. Microbiological processing was in accordance to the Clinical and Laboratory Standards Institute criteria [31]. Antimicrobials administration was categorized as empiric therapy, i.e., therapy administered 24 h prior to 48 h following the culture date (as long as there was no documentation that the attending physician was familiar prior with the result), and to main therapy, i.e., therapy administered 48 h following culture date (or prior if there was documentation that the attending physician was familiar with the result). Time to appropriate therapy was captured in days, from obtaining culture to the time that the first dose of drug with in vitro susceptibility to the offending pathogen was administered.

### Statistical Analyses

All analyses were executed with SPSS© (IBM^®^; V. 27.0, Armonk, NY, USA). Patients’ and offending pathogens’ characteristics and features are presented descriptively. The risk factors and outcomes for NGFGN HAUTI were queried with logistic and Cox regressions, respectively.

Logistic and Cox regressions were also used in order to analyze predictors and outcomes for empiric anti-Pseudomonal therapy for HAUTI. First it was queried among the entire cohort, and next among the cohort of patients who were managed empirically with beta-lactam agent/s only.

## Figures and Tables

**Table 1:** Pathogens of hospital-acquired urinary tract infections (HAUTI), Shamir Medical Center, 2017–2018.

Organism Type	Organism’s Name	Frequency	Valid Percent ^1^
Polymicrobial HAUTI		107	26
HAUTI-associated with bacteremia (i.e., same pathogen)		42	10.2
**List of offending organisms**			
Non-glucose fermenting Gram negatives (NGFGN)	*Pseudomonas aeruginosa*	62	14.9
	*Acinetobacter baumannii*	12	2.9
	*Stenotrophomonas maltophilia*	1	0.2
	*Achromobacter xylosoxidans*	1	0.2
	**Overall**	**75**	**18.2**
Glucose fermenting Gram negatives (i.e., GFGN)	*Escherichia coli*	130	31.4
	*Klebsiella pneumoniae*	85	20.6
	*Proteus mirabilis*	49	16.3
	*Providencia* species	10	2.3
	*Citrobacter* species	10	2.2
	*Morganella morganii*	4	0.9
	*Serratia* species	3	0.6
	*Klebsiella oxytoca*	2	0.4
Gram-positive bacteria	*Enterococcus* species	124	29.9
	*Streptococcus agalactiae*	4	0.8
**Extensively drug-resistant organisms (XDRO)**			
Carbapenem non-susceptible *Acinetobacter baumannii*		11	2.5
Carbapenem non-susceptible *Pseudomonas aeruginosa*		9	2.1
Carbapenem resistant Enterobacterales (CRE)		2	0.4
**Overall XDRO**		**23**	**5.6**

Note. HAUTI—hospital-acquired urinary tract infection; NGFGN—non-glucose fermenting Gram negatives; XDRO—extensively drug-resistant organism [24]. ^1^ Valid percent: percent after removing missing values from the denominator.

**Table 2:** Predictors and outcomes of hospital-acquired urinary tract infections (HAUTI) resulting from non-glucose fermenting Gram negatives (NGFGN), Shamir Medical Center, 2017–2018.

Parameter		NGFGN HAUTI (*n* = 75)		GFGN HAUTI (*n* = 338)		Statistics	
		Number	Percent	Number	Percent	OR (CI-95%)	*p*-Value
**Demographics**							
Age, years, median (IQR)		79 (70–84)		78 (67–85)			0.65
Age ≥ 65 years		64	85.3	275	81.8	1.2 (0.6–2.5)	0.47
**Male gender**		**42**	**56**	**137**	**40.8**	**1.8 (1.1–3)**	**0.016**
**Days from admission to HAUTI diagnosis, median (IQR)**		**15 (10–30)**		**10 (5–17.7)**			**<0.001**
Unit at HAUTI diagnosis	Medicine	45	60	225	67	0.74 (0.44–1.2)	0.25
	Surgery	19	25.7	57	17	1.6 (0.91–3)	0.09
	Gynecology (no Obstetrics enrolled)	0	0	4	1		>0.99
	Adult ICUs	11	14.9	50	14.9	0.98 (0.48–2)	0.96
**Chronic background medical statuses and condition** **s**							
Dependent functional status [23] in background		47	62.7	190	56.5	1.3 (0.8–2.1)	0.33
Altered cognition/consciousness in background		30	40	107	31.8	1.4 (0.8–2.4)	0.17
Charlson’s scores [22]	Combined Condition Score, median (IQR)	6 (4–8)		6 (5–8)			0.85
10-Years survival probability, percent, median (IQR)		2 (0–53)		2 (0–21)			0.77
Diabetes mellitus		29	38.7	164	49	0.65 (0.3–1)	0.1
Chronic kidney disease ^1^		15	20	89	26.5	0.69 (0.3–1.2)	0.24
Dementia		29	38.7	103	30.7	1.4 (0.8–2.4)	0.17
Hemi/paraparesis or hemi/paraplegia		7	9.7	46	13.7	0.64 (0.2–1.5)	0.3
**Chronic skin ulcers**		**16**	**21.3**	**41**	**12.2**	**1.9 (1–3.7)**	**0.039**
Malignancy (past and/or active)		17	22.7	69	20.5	1.1 (0.6–2)	0.68
Immunosuppression ^2^		12	16	51	15.2	1 (0.5–2.1)	0.85
**Had MDRO ^3^ isolated from the previous 2 years**		**24**	**32**	**53**	**15.8**	**2.5 (1.4–4.4)**	**0.001**
**Recent exposures to healthcare settings, procedures, environments**							
Residency at LTCF prior to current hospitalization		15	20	51	15.2	1.4 (0.7–2.6)	0.28
Recent (past 3 months) LTCF stay prior to current hospitalization		17	22.7	59	17.6	1.4 (0.7–2.5)	0.27
Recent hospitalization (past 3 months) in acute-care hospital		33	44	110	32.7	1.6 (0.97–2.6)	0.06
Weekly visits to outpatient clinics ^4^		4	5.3	7	2.1	2.6 (0.75–9.2)	0.11
**Has a permanent device ^5^ at admission**		**18**	**24**	**49**	**14.6**	**1.8 (1–3.4)**	**0.04**
**Had an invasive procedure ^6^ in the past 6 months**		**30**	**40**	**84**	**25**	**2 (1.1–3.3)**	**0.009**
**Antibiotics usage in the preceding 3 months ^7^**		**55**	**73.3**	**147**	**43.8**	**3.5 (2–6.1)**	**<0.001**
**Factors related to the urinary catheter**							
**Catheter in place at culture date or the day before ^8^**		**58**	**77.3**	**219**	**63**	**2 (1.1–3.6)**	**0.018**
≥2 days with catheter prior to the date of HAUTI diagnosis ^9^		54	72	198	60	1.7 (0.98–2.9)	0.053
**Number of days with catheter, median (IQR)**		**12.5 (5–30)**		**8 (3–20)**			**0.04**
**CAUTI cases ^10^**		**53**	**70.7**	**185**	**55.1**	**1.9 (1.1–3.3)**	**0.013**
Of the patients with catheters, the catheterization indication	**Chronic catheter**	**14**	**20.9**	**27**	**9.5**	**2.5 (1.2–5)**	**0.009**
	Post-surgery	10	15.4	31	11	1.42 (0.66–3)	0.36
	Accurate monitoring of urine output	38	56.7	192	67.8	0.7 (0.42–1.2)	0.22
	Acute retention	5	7.5	30	10.6	0.68 (0.25–1.8)	0.65
Catheter replacement at HAUTI onset		3	4.9	10	3.8	1.3 (0.3–4.9)	0.45
**Genitourinary tract abnormality**		**20**	**26.7**	**51**	**15.4**	**2 (1.1–3.6)**	**0.02**
Nephrolithiasis		3	4	11	3.3	1.2 (0.3–4.5)	0.48
Urine stent at HAUTI diagnosis		0	0	7	2.1	0.97 (0.96–0.99)	0.24
**Nephrostomy**		**6**	**8**	**4**	**1.2**	**7.2 (1.9–26)**	**0.003**
Urine procedure		5	6.7	12	3.6	1.9 (0.65–5.6)	0.18
**Acute illness indices**							
Clinical manifestations on the date of HAUTI	Fever	55	73.3	215	64	1.5 (0.8–2.7)	0.12
	Suprapubic tenderness	3	4	33	9.8	0.38 (0.1–1.3)	0.075
	Flank pain	1	1.1	5	1.5	0.899 (0.1–7.7)	0.7
	Urgency	2	2.7	14	4.2	0.6 (0.14–2.8)	0.67
	Frequency	2	2.7	9	2.7	1 (0.2–4.7)	0.61
	**Dysuria**	**6**	**8**	**103**	**30.7**	**0.2 (0.08–0.47)**	**<0.001**
Bacteremia (with the same pathogen)		6	8	36	10.7	0.73 (0.29–1.8)	0.48
Septic shock [25]		11	14.7	26	7.7	2 (0.9–4.3)	0.058
In ICU at culture date		13	17.3	56	16.7	1.8 (0.47–1.8)	0.88
Acute kidney injury ^11^		20	26.7	92	27.5	0.96 (0.5–1.6)	0.88
Altered consciousness at acute illness		40	53.3	158	47	1.2 (0.7–2.1)	0.32
Rapidly fatal McCabe [26]		17	22.7	54	16.1	1.5 (0.8–2.8)	0.17
**Empiric antimicrobial therapy**							
Days from culture to appropriate therapy, median (IQR) ^12^		2 (0–3.75)		1 (0–3)			0.08
Appropriate therapy in 48 h ^13^		30	41.1	173	53.2	0.61 (0.3–1)	0.061
**Outcomes**							
Died during current hospitalization		22	29.3	88	26.2	1.1 (0.6–2)	0.57
Died during 14 days after culture date		13	17.3	52	15.5	1.1 (0.5–2.2)	0.69
Died during 90 days after culture date		25	33.3	134	39.9	0.7 (0.4–1.2)	0.29
Among survivors of the index hospitalization only	**Length of stay from HAUTI to discharge, median (IQR)**	**12 (6–23)**		**8 (4–15)**			**0.01**
	Functional status deterioration at discharge following the HAUTI	28	52.8	116	47.2	1.2 (0.69–2.2)	0.45
	Discharge to LTCF (only among patients who were admitted to the index hospitalization from home)	22	46.8	86	38.4	1.4 (0.7–2.6)	0.28
	*Clostridioides difficile* isolation in 90 days following the HAUTI	3	5	12	4.2	1.2 (0.33–4.4)	0.76
	Additional hospitalization in 3 months	21	38.9	88	35.5	1.1 (0.6–2.1)	0.63

Note. NGFGN—non-glucose fermenting Gram negatives; GFGN—glucose fermenting Gram negatives; HAUTI—hospital-acquired urinary tract infection; IQR—interquartile range; ICU—intensive care unit; CAUTI—catheter-associated urinary tract infection; LTCF—Long-term care facility; MDRO—Multi-drug-resistance organism. ^1^ Estimated glomerular filtration rate less than 60 mL/min for three or more months. ^2^ The patient was considered immunosuppressed if he had any of the following: glucocorticoids exposure for ≥48 h in the past month, exposure in the past 3 months to chemotherapy, radiotherapy or immunomodulators (e.g., ant-TNF-α therapy), HIV carrier, past bone marrow or solid organ transplantation. ^3^ Any isolation from any site (i.e., not necessarily blood) in the past 2 years of multi-drug-resistant pathogens: methicillin-resistant *Staphylococcus aureus* (MRSA), vancomycin-resistant *Enterococcus*, extended-spectrum beta-lactamase or carbapenemase producing Enterobacterales (ESBL or CRE, respectively), *Acinetobacter baumannii*, or *Pseudomonas aureginosa.* ^4^ Patient attended an outpatient clinic at least weekly in the past three months. ^5^ Permanent devices include any of the following: chronic urinary catheters, tracheotomies, chronic tunneled central lines (e.g., PICC line), orthopedic external fixators, implanted defibrillators, or pacemakers, drains of any sort (e.g., genitourinary stoma). Prosthetic heart valves, prosthetic joints, and urologic or coronary internal stents were not considered a permanent device. ^6^ Any type of surgery (minor to major) or invasive procedure (e.g., endoscopy, percutaneous intervention) in the past 6 months. ^7^ At least two doses of any antibiotic in the past 3 months preceding HAUTI. ^8^ Patients with urinary catheter on the day the culture was taken or the day before, including patients with chronic catheters. ^9^ Patients for which the catheter was in place for at least two calendar days. ^10^ A CAUTI per CDC definitions [27]. ^11^ Any acute rise in creatinine level (>1.7 mg/dL, or 50% of baseline creatinine), or drop in estimated GFR by >50%. ^12^ The number of days from culture to initiation of “appropriate” antimicrobial therapy, implying the administration of an agent with activity per the in vitro microbiology laboratory report vs. the index pathogen. ^13^ Patient received “appropriate” antibiotic (as depicted above) in the first 48 h following the positive culture.

**Table 3:** Sub-analysis of patients with HAUTI who received a beta-lactam-only regimen.

Parameter		Empiric Beta-Lactam Regimen with Anti-Pseudomonals (*n* = 77)		Empiric Beta-Lactam Regimen with No Anti-Pseudomonals (*n* = 122)		Statistics	
		Number	Valid Percent *	Number	Valid Percent *	OR (CI-95%)	*p*-Value
**Demographics**							
Age, years, median (IQR)		77 (67–83)		79 (65–85)			0.5
Elderly (age ≥ 65 years)		68	88.3	96	78.7	2 (0.9–4.6)	0.08
Male gender		39	50.6	55	45.1	0.8 (0.4–1.4)	0.4
**Days from admission to HAUTI, median (IQR)**		**11 (6–19)**		**8 (4–14)**		**0.002**	
Unit at HAUTI diagnosis	Medicine	46	59.7	85	69.7	0.6 (0.3–1.1)	0.15
	Surgery	13	16.9	24	19.7	0.8 (0.3–1.7)	0.6
	Gynecology (i.e., no Obstetric)	0	0	3	2.5		>0.99
	**Adult ICU**	**18**	**23.4**	**10**	**8.2**	**3.4 (1.4–7.8)**	**0.002**
**Chronic background conditions and medical status**							
**Dependent functional status** [23]		**50**	**64.9**	**59**	**48.4**	**1.9 (1–3.5)**	**0.02**
Altered cognition/consciousness		23	29.9	35	28.7	1 (0.5–1.9)	0.8
Charlson’s scores [22]	Combined Condition Score, median (IQR)	6 (5–9)		6 (4–8)		0.2	
	10-Years survival probability, percent, median (IQR)	2 (0–21)		2 (0–53)		0.37	
Diabetes mellitus		41	53.2	59	48.4	1.2 (0.6–2.1)	0.5
Chronic kidney disease ^1^		28	36.4	29	23.8	1.8 (0.9–3.4)	0.056
Dementia		22	28.6	31	25.4	1.1 (0.6–2.2)	0.6
Hemiparesis/paraparesis, hemiplegia/paraplegia		11	14.3	10	8,2	1.8 (0.7–4.6)	0.17
Chronic skin ulcers		10	13	9	7.4	1.8 (0,7–4.8)	0.19
Malignancy (past and/or active)		20	26	26	21.3	1.2 (0.6–2.5)	0.4
Immunosuppression ^2^		9	11.7	17	13.9	0.8 (0.3–1.9)	0.6
Known MDRO ^3^ carrier		11	14.3	15	12.3	1.1 (0.5–2.7)	0.6
**Recent exposures to healthcare settings, procedures, environments**							
Residency at LTCF prior to hospitalization		14	19.5	18	14.8	1.3 (0.6–2.9)	0.3
Recent (past 3 months) LTCF stay prior to hospitalization		17	22.1	20	16.4	1.4 (0.7–2.9)	0.3
**Recent hospitalization (past 3 months) in an acute-care hospital**		**35**	**45.5**	**37**	**30.3**	**1.9 (1–3.4)**	**0.03**
Weekly visits to outpatient clinic ^4^		3	3.9	3	2.5	1.6 (0.3–8)	0.4
Permanent device ^5^ on admission		10	13	21	17.2	0.7 (0.3–1.6)	0.42
Invasive procedure ^6^ in the past 6 months		24	31.2	28	23	1.5 (0.8–2.8)	0.19
**Antibiotics usage in the preceding 3 months ^7^**		**43**	**55.8**	**45**	**36.9**	**2.1 (1.2–3.8)**	**0.009**
**Factors related to urinary catheter**							
Catheter in place at culture date or the day before ^8^		52	68.4	78	64.5	1.1 (0.6–2.1)	0.5
**Number of days with catheter, median (IQR)**		**13 (5–24)**		**9 (3–18)**		**0.05**	
**CAUTI cases ^9^**		**51**	**66.2**	**63**	**51.6**	**1.8 (1–3.3)**	**0.04**
Catheterization indication (only among patients with catheters)	Chronic catheter	10	14.5	12	11.7	1.2 (0.5–3.1)	0.5
	Post-surgery	11	15.9	9	8.7	1.9 (0.7–5)	0.14
	Accurate monitoring of urine output	44	63.5	67	65	0.9 (0.5–1.7)	0.8
	Acute retention	4	5.8	14	13.6	0.3 (0.1–1.2)	0.12
Catheter replacement at HAUTI onset		3	5	4	4.2	1.2 (0.2–5)	0.5
Genitourinary tract abnormality		14	18.2	18	15	1.2 (0.5–2.7)	0.5
Nephrolithiasis		2	2.6	3	2.5	1 (0.1–6.4)	0.6
Urinary stent/s		2	2.6	1	0.8	3.2 (0.2–36)	0.3
Nephrostomy		4	5.2	1	0.8	6 (0.7–60)	0.07
Recent invasive urinary procedure		4	5.2	4	3.3	1.6 (0.3–6)	0.3
**Acute illness indices**							
Clinical manifestations at HAUTI diagnosis	**Fever**	**69**	**90**	**72**	**59**	**5.9 (2.6–13)**	**<0.001**
	Suprapubic tenderness	3	3.9	11	9	0.4 (0.1–1.5)	0.16
	Flank pain	1	1.3	2	1.6	0.7 (0.07–8.8)	0.8
	Urgency	1	1.3	7	5.7	0.2 (0.02–1.7)	0.12
	Frequency	0	0	4	3.3	0.1	
	**Dysuria**	**10**	**13**	**32**	**26.2**	**0.4 (0.2–0.9)**	**0.02**
Bacteremia (with the same pathogen)		10	13	18	14.8	0.8 (0.4–2)	0.7
Septic shock [25]		8	10.4	7	5.7	1.9 (0.6–5)	0.2
**In ICU at culture date**		**19**	**24.7**	**12**	**9.8**	**3 (1.3–6.6)**	**0.005**
Acute kidney injury ^10^		24	31.2	34	28.1	1.1 (0.6–2.1)	0.6
Altered consciousness at acute illness		39	50.6	49	40.2	1.5 (0.8–2.7)	0.14
**Rapidly fatal McCabe** [26]		**18**	**23.4**	**15**	**12.3**	**2.1 (1–4.6)**	**0.04**
**Outcomes**							
Appropriate therapy administered in less than 48 h ^11^		52	69.3	66	56.9	1.7 (0.9–3.1)	0.08
**NGFGN HAUTI eventually diagnosed**		**15**	**19.5**	**11**	**9.1**	**2.4 (1–5.5)**	**0.035**
**Died during current hospitalization**		**23**	**29.9**	**20**	**16.4**	**2.1 (1–4)**	**0.02**
Died in 14 days		12	15.6	21	17.2	0.8 (0.4–1.9)	0.76
Died in 90 days		33	42.9	53	43.4	0.9 (0.5–1.7)	0.9
**Total length of stay, days, median (IQR)**		**25 (16–44)**		**16 (11–34)**		**<0.001**	
Among survivors of the index hospitalization only	Length of stay from HAUTI to discharge, days, median (IQR)	11 (6–17)		8 (4–14)		0.17	
	**Functional status deterioration**	**30**	**55.6**	**39**	**38.2**	**2 (1–3.9)**	**0.03**
	**Discharge to LTCF (only among patients who were admitted to the index hospitalization from home)**	**24**	**49**	**29**	**31.2**	**2.1 (1–4.3)**	**0.037**
	Acute *Clostridioides difficile* infection in 90 days following HAUTI	4	5.6	5	4.8	1.1 (0.3–4.6)	0.5
	**Additional hospitalization in 3 months**	**58**	**37.4**	**32**	**31.7**	**2 (1.01–3.9)**	**0.044**

Note. NGFGN—non-glucose fermenting Gram negatives; HAUTI—hospital-acquired urinary tract infection; IQR—interquartile range; ICU—intensive care unit; CAUTI—catheter-associated urinary tract infection; LTCF—Long-term care facility; MDRO—Multi-drug-resistance organism. * after excluding the missing values from the denominator. ^1^ Estimated glomerular filtration rate less than 60 mL/min for three or more months. ^2^ The patient was considered immunosuppressed if he had one of the following: glucocorticoids exposure for ≥48 h in the past month, or exposure in the past 3 months to chemotherapy, radiotherapy or immunomodulators (e.g., ant-TNF-α therapy), HIV carrier, past bone marrow or solid organ transplantation. ^3^ Any isolation from any site (i.e., not necessarily blood) in the past 2 years of multi-drug-resistant pathogens: oxacillin-resistant *Staphylococcus aureus*, vancomycin-resistant *Enterococcus*, extended-spectrum beta-lactamase-producing Enterobacteriaceae (ESBL), *Acinetobacter baumannii*, or *Pseudomonas aureginosa.* ^4^ Patient attended an outpatient clinic on a weekly basis prior (3 months) to current hospitalization. ^5^ Permanent devices include any of the following: chronic urinary catheters (e.g., silicon-based catheters), tracheotomies, tunneled central lines (e.g., PICC line), orthopedic external fixators, implanted defibrillators, pacemakers, or drains of any sort (e.g., genitourinary stoma). Prosthetic heart valves, prosthetic joints, and urologic or coronary internal stents were not considered a permanent device. ^6^ Any type of surgery (minor to major) or invasive procedure in the past 6 months: e.g., permanent central line insertions, percutaneous endoscopic gastrostomy insertion, ascites paracentesis, percutaneous coronary intervention, or abscess drainage. ^7^ At least two doses of any antibiotic course in the past 3 months preceding HAUTI. ^8^ This signifies patients with urinary catheter on the day the culture was taken or on the day before, and also includes patients with chronic catheters. ^9^ CAUTI event was determined per CDC definition [27]. ^10^ Any acute rise in the creatinine level (>1.7 mg/dL, or 50% of baseline creatinine), or drop in estimated GFR by >50%. ^11^ Patient received appropriate antibiotic per in vitro susceptibility, in the first 48 h from culture date.

## Data Availability

Not applicable.
